# ANXA2 Protein and Its Role in Neurodegeneration Processes

**DOI:** 10.3390/life15030402

**Published:** 2025-03-04

**Authors:** Suzanna A. Partevian, Petr A. Slominsky, Maria I. Shadrina, Anelya Kh. Alieva

**Affiliations:** National Research Centre “Kurchatov Institute”, 2 Kurchatova Sq., 123182 Moscow, Russia; paslominsky@bk.ru (P.A.S.); maria.i.shadrina@yandex.ru (M.I.S.); anelja.a@gmail.com (A.K.A.)

**Keywords:** ANXA2, neurodegeneration, tauopathies, Parkinson’s disease, Alzheimer’s disease

## Abstract

ANXA2 is a multifunctional member of the annexin protein family, implicated in vesicular transport, antioxidant defense, and actin remodeling. Its role in oncogenesis is actively investigated, notably in glioblastoma, astrocytoma, and breast cancer. However, a growing body of literature explores ANXA2’s involvement in neurodegenerative processes. The evidence suggests a potential contribution of ANXA2 to the pathogenesis of primary and secondary tauopathies, as well as Parkinson’s disease. It is crucial to note that the majority of these findings are correlative and necessitate further experimental validation. This review therefore presents a comprehensive analysis of data pertaining to ANXA2’s involvement in various cellular processes, the disruption of which contributes to neurological pathologies.

## 1. Introduction

Neurodegenerative diseases (NDs), such as Alzheimer’s disease (AD) and Parkinson’s disease (PD), are a significant public health concern. NDs are characterized by common metabolic alterations, including protein folding and degradation disruption, oxidative stress, mitochondrial dysfunction, vesicle transport impairment, and neuroinflammatory processes. NDs are characterized by common metabolic alterations, including protein folding and degradation disruption, oxidative stress, mitochondrial dysfunction, vesicle transport impairment, and neuroinflammatory processes [1].

Annexins play an important role in these processes. Proteins encoded by these genes are multifunctional, Ca^2+^-dependent, and phospholipid-binding. They participate in endocytosis, exocytosis, membrane remodeling, regulation of actin dynamics, regulation of mRNA expression levels, and many other processes [2]. Annexins play an important role in the normal and pathological functioning of the nervous system. Normally, they contribute to neuronal development, influencing differentiation and proliferation. In pathological conditions associated with impaired cell proliferation, annexins are implicated in the progression of tumors such as glioma and astrocytoma. In NDs, altered annexin expression can exacerbate disease progression [3]. The most frequently studied annexin proteins in normal and pathological nervous system development are ANXA1, ANXA2, and ANXA5. For example, ANXA1 has been shown to participate in neuroregeneration and maintaining the integrity of the blood–brain barrier. This protein may be involved in both AD development and various ophthalmological disorders [3,4].

ANXA5 is involved in the development of cortical neurons and the protection of glial cells [3]. Recently, there has been a growing body of research focused on the function of ANXA2 in NDs. The available studies are mainly focused on the role of this protein in the pathogenetic mechanisms of PD and AD. For example, ANXA2 may indirectly participate in axon growth, β-amyloid catabolism, and α-synuclein internalization [5,6,7]. In addition, ANXA2, by interacting with partner proteins such as PTEN and EGFR, may be involved in the PI3K/Akt signaling pathway, which is important in the pathogenesis of NDs [8,9].

This review provides an analysis and systematization of key studies related to both the normal function of ANXA2 and its involvement in metabolic processes, alterations of which contribute to the development of NDs.

## 2. The Structure of the ANXA2 Protein

**Figure 1:**
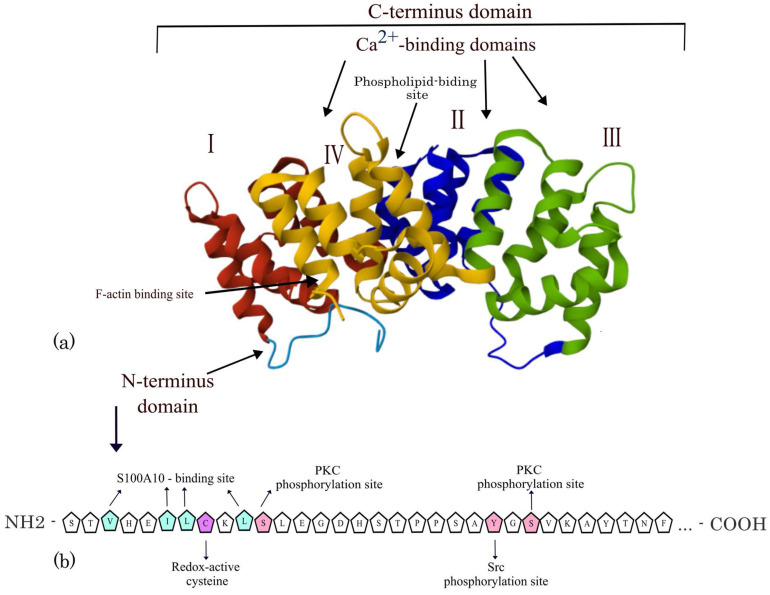
(**a**) The structure of the ANXA2 protein (pdb: 4X9P). The blue color indicates the N-terminal domain. Homologous motifs of the C-terminal domain are indicated in red, yellow, pink, and dark blue and are designated by Roman numerals [15]. (**b**) Primary structure of the N-terminal domain. Modified from [13,16].

Annexin A2 (ANXA2) is a multifunctional protein belonging to the annexin family with a molecular weight of 39 kDa [10]. The canonical form of ANXA2 consists of 339 amino acids, which form two domains: a variable head N-terminal domain and a core constitutive C-terminal domain resistant to proteases (Figure 1). Some authors divide the C-domain into two subdomains: the carboxyl and the core subdomains [11,12,13,14].

The C-terminal domain consists of four homologous motifs (Figure 1, each motif is highlighted in a different color). Each of these motifs contains approximately 70 amino acids. Each motif is made of five α-helices. Four of these helices are arranged in parallel, while one is positioned nearly perpendicular to the others [10,13,14]. Motifs II, III, and IV are characterized by the presence of calcium ion Ca^2+^-binding sites [17]. Additionally, the IV motif of this domain contains a phospholipid-binding site, involving the amino acid residues Arg-273 and Arg-284 [10].

The N-terminal domain consists of sites of post-translation modifications, such as acetylation and phosphorylation. These modifications affect the functional activity of ANXA2. Notably, acetylation of ANXA2 is required for its interaction with the S100A10 protein, leading to the formation of the AIIt complex (102 kDa). This complex is a heterotetramer composed of two S100A10 proteins (2 × 12 kDa) and two ANXA2 proteins (2 × 39 kDa) [18]. The phosphorylation sites serve as targets for SRC kinase and protein kinase C. This domain also harbors a binding site for the tissue plasminogen activator (tPA) [10,11,12].

It is important to note that ANXA2 can function both as a monomer and as the AIIt heterotetrameric complex within cells. As a monomer, ANXA2 plays a role in cellular antioxidant defense, the proteolysis of heparin, and the biogenesis of vesicles and early endosomes [11,19]. In contrast, as part of the AIIt complex, ANXA2 exhibits altered biological activity and participates in exocytosis, membrane structure repair, and the biogenesis of late endosomes [11,19]. Given that neurodegeneration involves disruptions in mechanisms related to transport and the functioning of the antioxidant system, this review focuses particularly on the involvement of ANXA2 in these processes.

## 3. Functions of the ANXA2 Protein

### 3.1. Role of ANXA2 in Exocytosis

**Figure 2:**
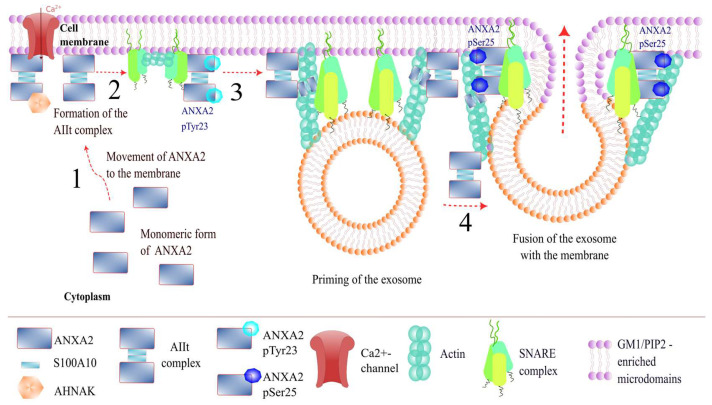
Participation of ANXA2 in exocytosis. 1. Movement of ANXA2 to the membrane to form the AIIt complex 2. Phosphorylation of ANXA2 in the AIIt complex by Tyr23. 3. Dephosphorylation of ANXA2, polymerization of actin. 4. Phosphorylation of ANXA2 by Ser25, vesicle fusion.

ANXA2 may participate in Ca^2+^-regulated exocytosis, leading to the secretion of various neurotransmitters and hormones. Notably, when involved in exocytosis, the ANXA2 protein undergoes various post-translational modifications aimed at interacting with the SNARE complex and actin. In vitro studies on cultured chromaffin cells have demonstrated ANXA2’s involvement in the exocytosis process at different stages: docking, priming, and the actual secretion of vesicle content (Figure 2) [20].

Before stimulation of chromaffin cells with nicotine, ANXA2 exists as a monomer in the cytoplasm of cells. After stimulation, ANXA2 translocates to the plasma membrane, where it forms a heterotetrameric AIIt complex, associating with SNARE complex proteins and monomeric actin [20,21]. Subsequently, ANXA2 within this complex is phosphorylated by Src kinase on Tyr23 and participates in the formation of PIP2/GM1-enriched lipid microdomains on the plasma membrane [21,22]. PIP2 (phosphatidylinositol-4,5-bisphosphate) and GM1 (ganglioside) are components of the plasma membrane and are necessary for the formation of exocytosis sites, the assembly of the SNARE complex on them, and the subsequent SNARE-mediated docking of vesicles to the plasma membrane [21,22]. Next, ANXA2 is dephosphorylated at Tyr23, which contributes to the formation of actin polymers on the plasma membrane [20]. Actin bundles are required for stabilizing exocytotic vesicles during their interaction with the plasma membrane [22]. After these interactions, ANXA2 is phosphorylated on Ser25 and participates in the fusion of vesicles with the plasma membrane. This is followed by the release of the vesicle contents through the contraction of actin bundles. The final stage of exocytosis involves the phosphorylation of ANXA2 at Ser11 and dissociation of the tetrameric complex [20].

The role of the AHNAK/ANXA2/S100A10 complex (1:2:2, AHNAK-AIIt) in exocytosis deserves special attention. This complex is present in GABAergic and glutamatergic neurons and may be involved in the formation of L-type calcium channels—VGCC. VGCC channels play a key role in exocytosis, as they allow calcium ions, which are necessary to initiate this process, to enter the cell [23]. The results obtained from experiments in AHNAK protein knockout mice indicate the importance of the AHNAK-AIIt complex. In such mice, a decrease in calcium influx through VGCC channels in GABAergic and glutamatergic neurons was observed. Thus, the AHNAK-AIIt complex may be one of the participants in Ca^2+^-dependent exocytosis in GABAergic and glutamatergic neurons [11,24].

The AHNAK-AIIt complex is also involved in the formation of enlargosomes. Enlargosomes are small non-secretory vesicles that increase the surface area of the plasma membrane during Ca^2+^-dependent exocytosis [25]. In a resting state, the AIIt complex covers the surface of enlargosomes. Conversely, AHNAK is located on the luminal surface of enlargosomes. Upon an increase in Ca^2+^ ion concentration, AHNAK is transported to the outer membrane of the enlargosome and localizes with the AIIt complex [26,27,28]. Subsequently, AHNAK, in conjunction with the AIIt complex, interacts with SNARE and VAMP8 proteins, and therefore may participate in the process of exocytosis [11,27,29,30].

Summarizing the above, it can be supposed that ANXA2 is involved in exocytosis through several pathways. On the one hand, ANXA2, as part of the AIIt complex, facilitates the interaction of vesicles with the plasma membrane, and participates in the formation of lipid microdomains on the plasma membrane. On the other hand, ANXA2 regulates the process of Ca^2+^-dependent exocytosis through the formation of the AHNAK–AIIt complex and is involved in the formation of enlargosomes.

### 3.2. Role of ANXA2 in Endocytosis

**Figure 3:**
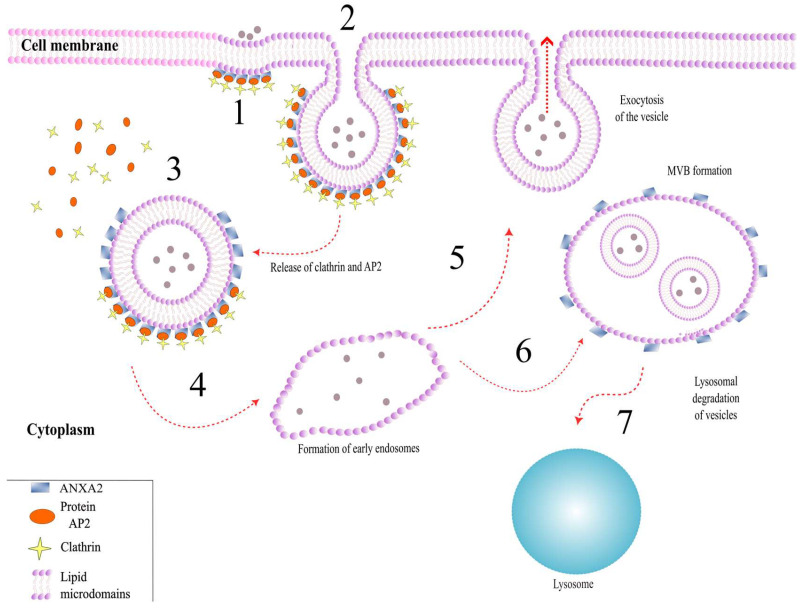
Participation of ANXA2 in endocytosis. 1. Initiation of endocytosis. Interaction of ANXA2, AP-2, and clathrin. 2. Bending of the membrane of early endosomes. 3. Cleavage of the endosome. Removal of clathrin and AP2 protein from the surface of the endosome. 4. Formation of early endosomes. 5. Vesicle recycling, exocytosis. 6. Formation of a multivesicular body. 7. Lysosomal degradation of vesicles and their contents.

Currently, there are numerous studies indicating the involvement of the monomeric form of ANXA2 in clathrin-dependent endocytosis. It is assumed that in this process, ANXA2 can interact with cholesterol and PIP2, which allows ANXA2 to localize on the inner surface of the plasma membrane of cells [11]. This interaction leads to the formation of lipid microdomains and the participation of ANXA2 in almost all stages of endocytosis (Figure 3) [10,31].

At the stage of initiation of endocytosis, ANXA2 interacts with the *μ2* subunit of the clathrin adaptor complex AP2 through its N-terminal region [32]. Subsequently, AP2 facilitates the recruitment of other endocytosis initiation factors, as well as the assembly of the clathrin complex [33]. At this stage, a clathrin-coated vesicle begins to form. Next, ANXA2 participates in the bending of the plasma membrane through the formation of specific lipid domains. This leads to the detachment of the vesicle from the cell membrane [34]. It is also hypothesized that, at this stage, temporary polymerization of F-actin occurs with the involvement of ANXA2 [35].

After detaching from the membrane, the vesicle moves into the cytoplasm, where clathrin and AP2 are released. Then, the vesicle fuses with the early endosome [31]. There is evidence suggesting that ANXA2 can interact with early endosome proteins—APPL1 and APPL2. Based on this, ANXA2 can be considered a marker for early endosomes, along with APPL1 and APPL2 [36].

ANXA2 also participates in the maturation of early endosomes by remodeling their membrane [37]. It is known that ANXA2 localizes to PIP2-enriched membranes of early endosomes and can act as an adapter between early endosomes and F-actin [22]. At this stage, ANXA2 acts as a nucleation factor for F-actin in conjunction with Spire1 and Arp2/3 proteins, and also localizes to the growing end of actin [38,39]. Early endosomes, interacting with polymeric actin, are transported from the plasma membrane into the cytoplasm, fuse and form multivesicular bodies, or are recycled, depending on the needs of the cell [40]. However, to date, the role of ANXA2 in choosing the path an endosome will take has not been proven.

Currently, significant interest in studying endosome recycling and maturation has focused on the role of the AIIt complex in this process. The formation of the AIIt complex is necessary if the endosome is to be recycled [41]. However, there are conflicting data regarding the localization of AIIt on the membrane of late endosomes. In the work of E. Morel and J. Gruenberg (2007), it was shown that AIIt is not required if the endosome follows the degradation pathway [42]. However, more recent studies show that AIIt is localized on the membrane of late endosomes that follow the lysosomal degradation pathway [43,44]. It is noteworthy that phosphorylation of ANXA2 at Tyr23 within the AIIt complex during the formation of multivesicular bodies is necessary [11,45].

Therefore, research highlights the important role of ANXA2 at all stages of endocytosis. However, further study is required to investigate the formation and function of the AIIt complex in the later stages of this process.

### 3.3. Role of ANXA2 in Antioxidant Protection of Cell

Among the entire annexin family, ANXA2 is unique in that it contains redox-sensitive cysteines—Cys8, Cys132, Cys261, and Cys334. The reactive residues involved in the regulation of cellular oxidative stress in ANXA2 are Cys8 and Cys132.

In a study by D.M. Sullivan et al. (2000) devoted to the study of protein glutathionylation in response to oxidative stress, it was first demonstrated that ANXA2 is a target when exposed to reactive oxygen species (ROS). Notably, this is the only study showing that glutathionylation occurred at the amino acid residue Cys9 of ANXA2 [46]. However, in all subsequent studies addressing the role of ANXA2 in oxidative stress, it has been shown that Cys8 of ANXA2 is the reactive residue when exposed to oxidative stress. This discrepancy in the protein sequence is likely due to differences in annotation.

When the ANXA2 protein is expressed on *E. coli* bacterial culture, it has been shown that Cys8 and Cys132 can undergo glutathionylation under oxidative stress. This reaction causes ANXA2 to lose its functions, preventing it from binding F-actin and phospholipids. However, treatment with glutaredoxin restores these functions [47].

The role of ANXA2 in cellular antioxidant defense has been investigated in more detail. Thus, Cys8 can be directly oxidized by H_2_O_2_ and then reduced by the thioredoxin system, demonstrating antioxidant properties [10]. ANXA2 can also act as an antioxidant through interaction with the phosphatase and tensin homolog (PTEN) via its Cys8 residue in the PI3K/Akt pathway, as follows. Upon activation of the PI3K/Akt pathway through interaction of epidermal growth factor (EGF) with its receptor (EGFR), activation of NADPH oxidase (NOX) occurs, leading to H_2_O_2_ production. ANXA2 regulates PTEN, which is an inhibitor of the PI3K/Akt pathway. This interaction can enhance PTEN activity, inhibit the PI3K/Akt pathway, and thus inactivate H_2_O_2_ production [9].

The involvement of ANXA2 in antioxidant defense is supported by studies in NOD-SCID mice, a strain with immunodeficiency and predisposition to nonobese diabetes. Reduction in ANXA2 expression through siRNA delivery led to increased production of ROS and increased levels of oxidized proteins in liver and lung tissues, as well as activation of pro-apoptotic enzymes. Conversely, in various cancer cell lines (MCF-7, A549, HT1080, LLC, and 293T) with ANXA2 deficiency, elevated ROS levels, enhanced activation of pro-apoptotic kinases, and increased sensitivity to ROS were also observed [48].

Based on the above, it can be concluded that ANXA2 is a crucial participant in cellular antioxidant defense, acting as both a target (through interaction with glutathione) and an antioxidant (through direct interaction with H_2_O_2_ and regulation of the PI3K/Akt pathway). On one hand, due to the presence of cysteine residue Cys8 in its structure, ANXA2 undergoes glutathionylation, acting as a target for ROS. On the other hand, Cys8 inactivates H_2_O_2_ and is subsequently reduced by the thioredoxin system. Furthermore, ANXA2, in conjunction with PTEN, can act as an inhibitor of the PI3K/Akt pathway, which also leads to a decrease in the production of ROS.

## 4. Involvement of ANXA2 in the Development of Neurodegenerative Processes

Currently, there are numerous studies indicating the involvement of ANXA2 in the development of cancer. The data on the involvement of ANXA2 in cancer development are summarized in numerous reviews, including those focusing on glioblastoma and the function of the AIIt complex as a receptor for tPA and plasminogen [49,50]. Furthermore, there are several studies describing the participation of ANXA2 in the pathogenesis of NDs such as primary and secondary tauopathies and Parkinson’s disease (PD) [5,51].

### 4.1. The Involvement of ANXA2 in the Pathogenesis of Tauopathies

**Figure 4:**
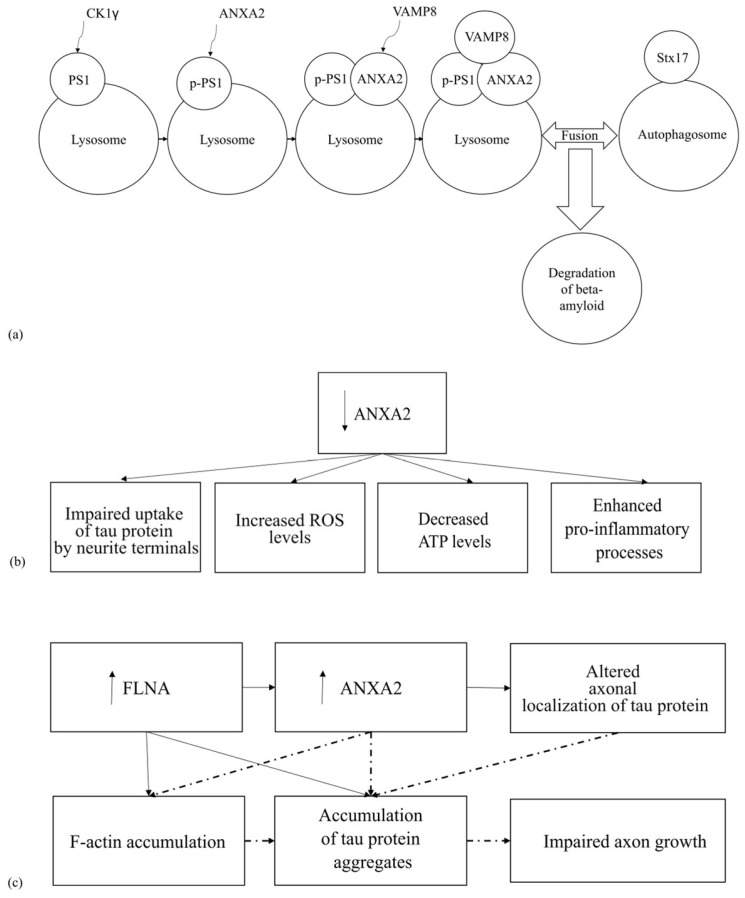
The involvement of ANXA2 in the pathogenesis of tauopathies. Solid arrows indicate experimental data; dashed lines indicate hypotheses. (**a**) The involvement of ANXA2 in the degradation of beta-amyloid; (**b**) the effects of ANXA2 downregulation on tauopathy pathogenesis; (**c**) the effect of FLNA and ANXA2 interaction on tauopathy pathogenesis.

Tauopathies are a group of diseases characterized by the accumulation of the tau protein in the central nervous system. These accumulations lead to the development of dementia and motor disorders [52]. Tauopathies are divided into two groups: primary and secondary tauopathies. Primary tauopathies include those diseases in which the tau protein aggregation predominates, while secondary tauopathies involve tau protein aggregation in addition to aggregation of other proteins. An example of a primary tauopathy is frontotemporal dementia with parkinsonism (FTDP-17), and an example of a secondary tauopathy is AD (Figure 4) [53].

Tau protein is involved in tubulin polymerization, microtubule assembly, and maintenance of their stability. Maintaining microtubule stability promotes axon growth [54]. It is known that tau protein can interact with the growth cone of axons. The interaction between the axon membrane and axonal localization of the tau protein is mediated by the protein ANXA2, possibly in complex with the protein S100A10. This complex maintains the integrity of microtubules in neuronal axons, which is necessary for axon growth. It is hypothesized that the mutant tau protein is unable to bind to membranes due to impaired interaction with ANXA2, leading to disruption of normal axon growth and, according to the authors, causing neurodegeneration in FTDP-17. Furthermore, downregulation of ANXA2 results in impaired sequestration of tau protein at axonal termini [5,55]. These data may indicate the importance of the tau protein-ANXA2 interaction for axonal tau localization.

It has previously been shown that the FLNA protein may be involved in the pathogenesis of various tauopathies [56,57]. It is also known that FLNA and ANXA2 are involved in altering the structure of F-actin, which may also be important for the pathogenesis of tauopathies in general [58]. To further investigate the role of the FLNA protein in the development of tauopathies, FLNA protein overexpression was investigated in vitro on N2a neuroblastoma cell culture and on HEK293 cell line, as well as in vivo in mice. In these in vivo and in vitro experiments, an increase in the concentration of insoluble tau protein and its mutant forms (P301L, V337M, and R406W) was observed [56,59]. Moreover, in vivo, studies have shown that FLNA overexpression leads to F-actin accumulation and slowed [56]. This accumulation results in increased levels of the 4R tau isoform, followed by its aggregation in neurons, oligodendrocytes, and astrocytes [56]. A subsequent independent study in the N2a cell line also observed increased ANXA2 expression upon FLNA overexpression [59]. The authors hypothesize that F-actin reorganization may, on the one hand, relieve spatial blockage of mRNA transcripts encoding the tau protein and ANXA2. On the other hand, this reorganization may facilitate the transport of mRNA transcripts encoding the tau protein and ANXA2 to translationally active sites [59].

Based on the described data, it is probable that an increase in the level of FLNA leads to slowed F-actin polymerization and increased ANXA2 protein levels, which in turn may result in the accumulation of insoluble tau protein and disruption of its axonal localization (Figure 4c). However, it is currently unknown exactly how this interaction occurs. The totality of the data described above suggests that both excessive lowering and increasing of the expression of the ANXA2 protein can lead to disruption of the normal functioning of neurons. On the one hand, there is a disruption of the binding of the tau protein to the membrane, and on the other hand, there is an accumulation of insoluble tau protein. It should be noted that these hypotheses require further experimental validation.

In AD, the formation of amyloid plaques is a key component of pathogenesis. These plaques can include the tau protein [60]. It is known that PSEN1 and tPA play a key role in the catabolism of amyloid plaques [61,62,63]. PSEN1 is part of the γ-secretase complex, which is involved in the degradation of type I transmembrane proteins. These proteins include amyloid precursor protein (APP), Notch, and β-catenin [64,65]. In addition, PSEN1 phosphorylated at Ser367 (PSEN1-Ser367) can interact with ANXA2 and participate in the degradation of βCTF (β-C-terminal fragment of APP) through autophagy. In this case, ANXA2 interacts with VAMP8 on the surface of the lysosome and promotes the binding of VAMP8 to syntaxin 17 on the surface of the autophagosome. Next, the autophagosome fuses with the lysosome and further degradation of βCTF occurs, leading to a decrease in β-amyloid (Aβ) levels (Figure 4a) [7,65].

Another important protein involved in β-amyloid metabolism and interacting with ANXA2 is tPA. tPA is capable of participating in the plasmin-induced degradation of insoluble Aβ-containing extracellular plaques, thereby potentially suppressing the amyloid etiology of AD [62]. The AIIt complex is known to act as a receptor for both tPA and plasminogen. This interaction leads to plasminogen activation and the formation of plasmin [66,67]. Plasmin, in turn, plays a role in Aβ degradation [62]. This suggests that the binding of tPA and plasminogen to the AIIt receptor may enhance plasmin-induced β-amyloid degradation. However, there is currently no compelling evidence supporting this theory.

In this context, it is impossible to ignore the review by P.K. Jayaswamy, which describes the potential consequences of tPA blockade by the factor PAI-1 [7]. The authors suggest that blocking tPA with PAI-1 disrupts the AIIt–tPA complex on the cell membrane, leading to impaired plasmin formation and plasmin-mediated degradation of Aβ1-42. They propose that, in response, ANXA2 translocates from the cytoplasm to the membrane to help restore the AIIt–tPA complex, which is crucial for plasmin generation and Aβ1-42 degradation. However, they also suggest that this tPA inhibition by PAI-1 leads to ANXA2 predominantly associating with EGFR, activating the p-STAT3 pathway, and subsequently promoting neuronal apoptosis [7]. Crucially, currently there is no experimental evidence directly linking these proposed events. Further research is thus necessary to validate the authors’ hypothesis.

Furthermore, in a study by Ye Lianmeng et al. focusing on identifying novel genes involved in AD pathogenesis, interesting data were obtained indicating that ANXA2 may play an important role in AD. Specifically, on SH-SY5Y neuroblastoma and HMC3 microglia cell cultures secreting Aβ-42, knockdown of ANXA2 resulted in increased levels of ROS, decreased ATP levels, enhanced pro-inflammatory processes, apoptosis, and cell death (Figure 4b) [68]. Conversely, overexpression of this protein was associated with activation of anti-inflammatory processes. The authors therefore conclude that ANXA2 may play a protective role in the development of AD-related pathological processes and represents a potential target for further research [68].

Published data suggest that ANXA2 may be involved in the development of tauopathy through multiple pathways [7,59,65,68]. On the one hand, ANXA2 participates in β-amyloid autophagy, leading to its degradation, and increased expression of ANXA2 may promote anti-inflammatory processes [65,68]. On the other hand, ANXA2 overexpression may disrupt tau protein localization [59]. Conversely, decreased ANXA2 expression may lead to tau protein accumulation, enhanced apoptosis, increased cell death, elevated levels of ROS, and activation of pro-inflammatory processes [68]. However, the currently available data are indirect and require further experimental validation.

### 4.2. The Involvement of ANXA2 in the Pathogenesis of Parkinson’s Disease

**Figure 5:**
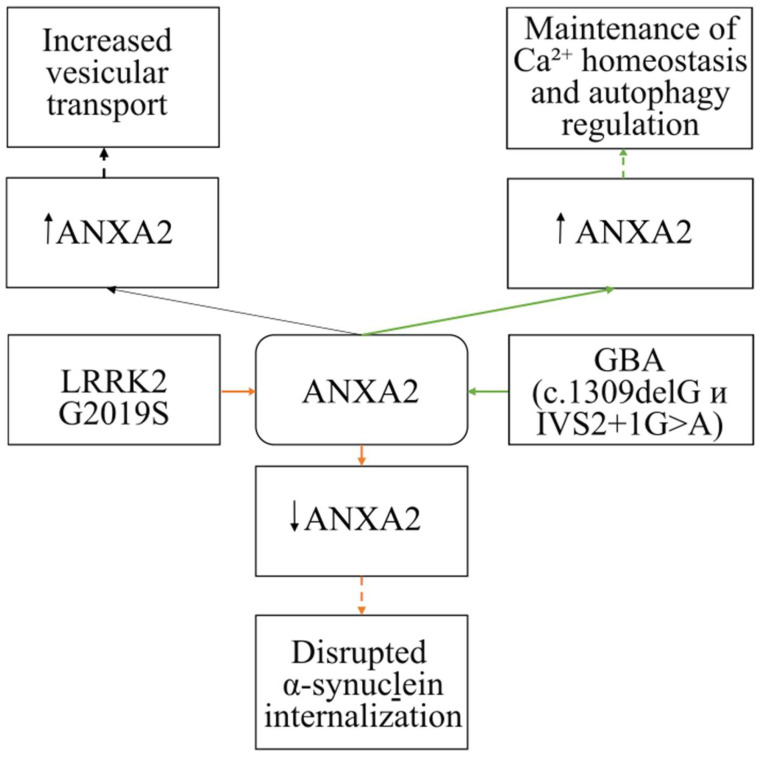
The involvement of ANXA2 in the pathogenesis of Parkinson’s disease. Orange arrows indicate the effect of the mutant form of LRRK2, green arrows indicate the effect of the mutant form of GBA. A black arrow indicates the effect of increased ANXA2 without any mutations. Solid arrows indicate experimental data; dashed lines indicate hypotheses.

There are numerous studies investigating the formation of α-synuclein aggregates in PD, as well as the mechanisms of lysosomal degradation impairment in PD associated with GBA gene mutations [69]. The available data suggest that ANXA2 may participate in the internalization of α-synuclein and the development of compensatory mechanisms in PD (Figure 5).

For example, the work of Streubel-Gallasch examined the uptake of fibrillar α-synuclein by astrocytes carrying the LRRK2 G2019S mutation [6]. It was observed that the mutant astrocyte line showed a reduced uptake of fibrillar α-synuclein, leading to decreased amounts of fibrillar protein within the cells. This same cell culture also exhibited reduced ANXA2 protein levels, which could contribute to the impaired uptake of fibrillar α-synuclein [6]. This hypothesis is based on ANXA2’s involvement in the process of endocytosis. Consequently, the authors conclude that the LRRK2 G2019S mutation leads to the accumulation of extracellular fibrillar α-synuclein due to impaired internalization, not through impaired lysosomal degradation. However, it remains unclear how LRRK2 and ANXA2 interact [6].

Our laboratory has also shown changes in *Anxa2* gene expression at the mRNA level in the brain and blood tissues of mice at early stages of PD. These findings may suggest the involvement of this protein in compensatory mechanisms in PD through enhanced vesicular transport [70].

Furthermore, ANXA2 may be involved in the pathogenesis of PD associated with GBA gene mutations. In a study of fibroblasts from PD patients who are heterozygous carriers of mutations in the GBA gene (c.1309delG and IVS2+1G>A), increased ANXA2 protein expression was detected. The authors suggest that the increased ANXA2 expression leads to the development of a compensatory mechanism in PD through maintaining calcium homeostasis and regulating autophagy [51].

Therefore, based on published data regarding the role of ANXA2 in NDs, it can be hypothesized that this protein may play a dual role in these pathologies. On the one hand, increased ANXA2 expression is observed concurrently with increased tau protein expression and disrupted intracellular distribution within nerve cells [59]. However, it remains unknown whether these processes are sequential or parallel. On the other hand, this increased expression leads to compensatory and anti-inflammatory processes [51,68,70]. This duality may be linked to the activation of various metabolic pathways involved in ND development. It should also be noted that decreased ANXA2 expression can lead to ROS accumulation, enhanced apoptosis, increased cell death, disrupted tau protein distribution, and impaired α-synuclein internalization [6,55,68]. Based on the available data, it can be concluded that ANXA2 may participate in the functioning of nerve cells under pathological conditions.

## 5. Conclusions

Currently, most research focuses on ANXA2’s involvement in cell division processes, primarily within the context of cancer. However, a growing body of work is exploring ANXA2’s role in neurodegenerative processes. In the nervous system, ANXA2 regulates vesicular transport by interacting with the lipid bilayers of both cellular and vesicular membranes. It also acts as both a target and an antioxidant in cellular antioxidant defense. Based on the current literature, ANXA2 appears to play a dual role in neurodegeneration. For instance, in tauopathies, and in particular Alzheimer’s disease, ANXA2 may participate in amyloid protein degradation, cellular antioxidant defense, and anti-inflammatory processes. However, ANXA2 may also contribute to disrupted tau protein interaction with axon terminals and intracellular aggregation. In PD, ANXA2 facilitates α-synuclein internalization, maintains Ca^2+^ homeostasis, and regulates vesicular transport. It is important to note that current studies primarily utilize cell cultures, yielding indirect evidence. Further validation in animal models and human studies is needed for a more detailed understanding. Such research could focus on identifying the molecular mechanisms by which ANXA2 contributes to nervous system development and neurodegeneration. Elucidating these mechanisms would enable the development of targeted therapies for NDs.

## Data Availability

No new data were created or analyzed in this study.

## Abbreviations

The following abbreviations are used in this manuscript:

NDs	Neurodegenerative diseases
PD	Parkinson’s disease
AD	Alzheimer’s disease
FTDP-17	Frontotemporal dementia with parkinsonism
ANXA2	Annexin A2
tPA	tissue plasminogen activator
ROS	reactive oxygen species
APP	amyloid precursor protein
